# The Detection of Mixed Infection with Canine Parvovirus, Canine Distemper Virus, and Rotavirus in Giant Pandas by Multiplex PCR

**DOI:** 10.3390/vetsci12020081

**Published:** 2025-01-23

**Authors:** Ai Liu, Wenyue Qiao, Rui Ma, Qigui Yan, Shan Zhao, Yifei Lang

**Affiliations:** 1 College of Veterinary Medicine, Sichuan Agricultural University, Chengdu 611130, China; 2 Chengdu National Agricultural Science and Technology Center, Chengdu 610213, China; 3 Sichuan Key Laboratory of Conservation Biology for Endangered Wildlife, Chengdu Research Base of Giant Panda Breeding, 1375 Panda Road, Chenghua District, Chengdu 610081, China

**Keywords:** canine parvovirus (CPV-2), canine distemper virus (CDV), giant panda rotavirus (GPRV), multiplex PCR, giant panda

## Abstract

The well-being and subsistence of giant pandas, an endangered species with a limited distribution, are currently threatened by a number of viruses, including canine parvovirus (CPV-2), canine distemper virus (CDV), and giant panda rotavirus (GPRV). To allow for timely intervention upon viral infection, it is necessary to execute rapid and accurate diagnosis of potential mixed viral infections. In the present study, we developed and validated a multiplex PCR (mPCR) approach for the detection of CPV-2, CDV, and GPRV infections. The results indicate that the method could selectively amplify the three viruses with high sensitivity and specificity, which are necessary attributes in clinical settings. Utilizing the established method, (sub)clinical giant panda samples were examined, and CPV-2, CDV, and GPRV were found in 19.72% (43 out of 218), 7.34% (16 out of 218), and 6.42% (14 out of 218) of the samples, respectively. Noticeably, mixed infections of two or three viruses were common, and this was generally observed in CDV- or GPRV-positive samples. Meanwhile, mPCR results were further validated with sequencing and the phylogenetic analysis of full-length sequences of viral genes. Taken together, our study provides an approachable assay which enables the quick detection of the three viruses mentioned above, which will benefit clinical diagnosis and laboratory epidemiological-based investigations of the giant panda population.

## Simple Summary

Virus infections pose significant threats to the health and survival of giant pandas, an endangered species, making rapid and accurate diagnosis crucial for effective management and conservation efforts. In this study, we developed a multiplex PCR (mPCR) method to detect canine parvovirus type 2 (CPV-2), canine distemper virus (CDV), and giant panda rotavirus (GPRV) in pandas. The mPCR approach was validated for its high sensitivity and specificity in identifying these viruses, including mixed infections. In a sample of 218 giant pandas, CPV-2, CDV, and GPRV were detected in 19.72%, 7.34%, and 6.42% of the cases, respectively, with over half of the positive samples showing mixed infections. The findings were further confirmed using sequencing and phylogenetic analysis. This method offers a valuable tool for clinical diagnosis and epidemiological studies, aiding in the protection of the giant panda population.

## 1. Introduction

Viruses are obligate intracellular parasites that can cause infectious diseases in different plant and animal species [1]. Unable to reproduce by themselves, their propagation is fully reliant on the metabolic and biosynthetic processes in living host cells [2,3]. To this end, viral infection often conveys detrimental consequences to their host and was even responsible for several major pandemics in human history. Meanwhile, viruses do not infect just one particular host; on the contrary, they tend to infect many host species within reach [4]. The host range expansion of viruses, mediated by cross-species transmission, provides a certain basis for the emergence of new diseases [5,6,7]. Such characteristics raise public health concerns and also pose an inevitable threat to a variety of endangered wild animals [8], such as giant pandas (*Ailuropoda melanoleuca*) [9,10].

**Figure 1:**
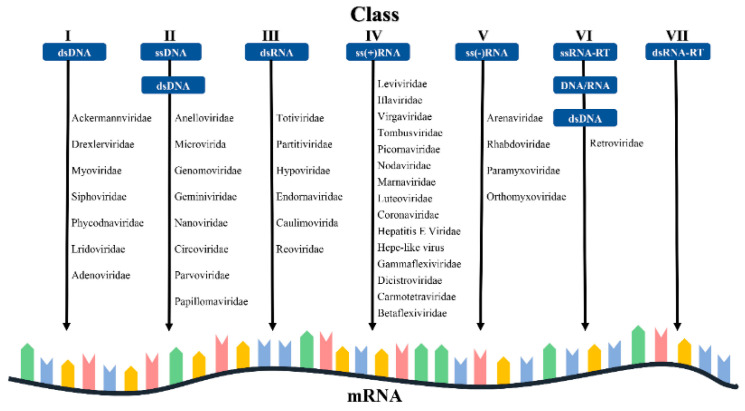
The Baltimore viral categorization system is used to show the various classes of viruses which infect giant pandas. I: Classes of double-stranded DNA viruses (dsDNA); II: classes of single-stranded DNA viruses (ssDNA); III: classes of double-stranded RNA viruses (dsRNA); IV: classes of positive-sense single-stranded RNA viruses (+ssRNA); V: classes of negative-sense single-stranded RNA viruses (-ssRNA); VI: classes of single-stranded RNA reverse transcription viruses (ssRNA-RT); VII: classes of double-stranded RNA reverse transcription viruses (dsRNA-RT).

As a vulnerable species listed by the International Union for the Conservation of Nature (IUCN), giant pandas appear as touristic icons and key representatives of biodiversity conservation. Today, giant pandas are facing many challenges, including habitat fragmentation, reproduction inconveniency, and numerous bacteria and virus infections [9,10,11,12,13]. Over the years, many viruses were confirmed to be able to infect giant panda, including canine parvovirus type 2 (CPV-2), canine adenovirus type 1 (CAV-1), canine distemper virus (CDV), giant panda rotavirus (GPRV), and influenza A virus (IAV). These lead to various and even fatal consequences [14,15,16,17,18]. Recently, with the advances in viromics research, traces of infection with more virus species were identified in giant pandas, as detailed in Figure 1 (Supplementary Table S1). Therefore, the etiological and epidemiological surveillance of viral infections that endanger the situation of giant pandas should be reinforced regularly to provide early warning for forthcoming threats.

In the present study, we confirmed the widespread infection and sporadic co-infection of CPV-2, CDV, and GPRV in giant pandas via the establishment and validation of a multiplex polymerase chain reaction (mPCR) assay. Our findings highlight the potential threat of these viruses to giant pandas, emphasize the importance of the timely and systematic surveillance of these viruses, and provide a fast and efficient method to achieve this goal.

## 2. Materials and Methods

### 2.1. Viruses and Cells

The CDV (strain A75/17, accession number: AF164967.1), CPV-2 (strain CPV-LZ2, accession number: JQ268284.1) and GPRV (strain CH-1, accession number: GU205762.1) samples used in this study were retrieved from the virus database in our laboratory. CDV and GPRV were propagated in porcine kidney epithelial cells (LLC-PK1), while CPV-2 was propagated in Crandell–Rees Feline Kidney (CRFK) cells. All cells were maintained in Dulbecco’s modified Eagle medium (DMEM, Gibco) supplemented with 10% fetal bovine serum (FCS), penicillin (100 IU/mL) and streptomycin (100 μg/mL). Porcine kidney epithelial (LLC-PK1) cells and Crandell–Rees Feline Kidney (CRFK) cells were purchased from ATCC (American Type Culture Collection, Rockefeller, Maryland, MD, USA).

### 2.2. Fecal Samples from Giant Panda

Giant panda fecal samples (*n* = 218) were collected from giant pandas at Chengdu Research Base of Giant Panda Breeding, China, between the year 2015 and 2020. Most of the sampled pandas are healthy, with the exception of approximately 10% of them, which displayed signs such as mild diarrhea, anorexia, and vomiting.

### 2.3. Primer Design

Primers for the multiplex PCR assay were designed based on the CDV N gene, the GPRV NSP1 gene, and the CPV-2 NS1 gene. Representative viral sequences of each virus were first analyzed with multiple-sequence alignment using MEGA X, and primers were selected from the conservative regions within each gene. The selected primer candidates were then analyzed with a BLAST search (NCBI) and the DNASTAR Lasergene software 15.1 package to omit homology with the giant panda genome or other viral sequences. To allow for further analysis of positive samples, sets of primers were also designed to amplify the corresponding full-length coding sequences of the CDV N gene, the GPRV NSP1 gene, and the CPV-2 NS1 gene. All the primers were synthesized in Sangon Biotech Company (Shanghai, China), and full sequence information is detailed in Table 1 and Table 2.

### 2.4. Preparation of Standard Plasmids

DNA sequences representing the ideal mPCR products of CDV (757 bp), GPRV (432 bp), and CPV-2 (293 bp) were synthesized and ligated into the pMD19-T Vector (TaKaRa). All standard plasmids were validated via conventional bidirectional sanger sequencing.

### 2.5. Establishment of the mPCR Method

The linearized standard plasmids (pMD-CDV-N, pMD-GPRV-NSP1, and pMD-CPV-2-NS) were utilized as architype templates to allow for the optimization of the PCR annealing temperature (Ta). The concentration of each primer was fixed at 100 nM, while each primer constituted 0.25 μL of the reaction volume (primer information is detailed in Table 1). The multiplex PCR assay was then conducted in a total volume of 20 μL per reaction, containing 10 μL of 2 × Taq Master Mix (Vazyme, P112-01), mixed template (pMD-CDV-N, pMD-GPRV-NSP1 and pMD-CPV-2-NS, 10 ng in total), primer mix, and ddH2O. The annealing temperatures for the mPCR reaction were optimized by ramping the temperature from 45 °C to 55 °C, while increasing by 1 °C in a sequential order. The procedure was as follows: 95 °C for 3 min, followed by 34 cycles of 95 °C for 15 s, the diversion Ta for 15 s, and cycles at 72 °C for 15 s, with a final extension at 72 °C for 5 min. After completion, 5 μL of the mPCR product was evaluated by 1.0% agarose gel electrophoresis.

### 2.6. Specificity and Sensitivity Analysis of the Optimized mPCR Assay

The optimized mPCR assay was used to examine the specificity of the multiplex PCR amplification of all possible combinations, namely, CDV, GPRV, CPV-2, CDV + GPRV, CDV + CPV-2, GPRV + CPV-2, and CDV + GPRV + CPV-2. The amplified target bands were then recovered, purified, and sequenced after ligation to pMD19-T Vector (TaKaRa) to confirm the specificity of the multiplex PCR. To further test the mPCR sensitivity, the concentrations of standard plasmids (pMD-CDV-N, pMD-GPRV-NSP1 and pMD-CPV-2-NS) were adjusted to 10 ng/μL as measured by a NanoDrop 2000 UV-vis spectrophotometer (Thermo Scientific, Waltham, MA, USA). The copy number was then calculated using the following formula: copies/μL= NA (copies/mol) × concentration (g/μL)/MW (g/mol). Moreover, each standard plasmid was serially diluted 10-fold, starting from 1 × 10^8^ to 1 × 10^1^ copies/μL with ddH2O. The plasmids of each dilution were then used as templates for the sensitivity testing of multiplex PCR.

### 2.7. Viral Nucleic Acid Extraction and Reverse Transcription

Total nucleic acid was extracted from giant panda fecal samples using a DNA/RNA extraction kit (Vazyme, RM201-02) and Stool DNA Isolation Kit (Foregene, DE-05713). This was performed according to the manufacturer’s protocol. The extracted total nucleic acids were divided into two portions; one was used for the amplification of the DNA viruses (CPV-2), and the other was used for the reverse transcription (RT) and amplification of the RNA viruses (CDV and GPRV). The RNA extracted from samples was reverse-transcribed to cDNA using the 2×RT OR-EasyTM Mix (Foregene, RT-01022) according to the manufacturer’s instructions. The cDNA and DNA were stored at −20 °C until PCR amplification, while the isolated RNA was stored at −80 °C.

### 2.8. Evaluation of Clinical Samples

A total of 218 panda fecal samples were collected and stored at −80 °C until use. The established multiplex PCR method was then used for virus detection using the isolated DNA and cDNA samples, which were obtained as described above. After mPCR amplification and 1.0% agarose gel electrophoresis, the infection rate was calculated and evaluated.

### 2.9. Extensive Full-Length Viral Gene Sequencing and Phylogenetic Analysis

To provide further detailed information about the positive samples, the DNA of the DNA virus (CPV-2) and the cDNA of the RNA viruses (CDV, GPRV) of mPCR-positive samples were extracted for PCR amplification. Sequence information about viral structural proteins, namely CDV-H, GPRV-VP3, and CPV-2-VP2, was obtained with the primer pairs listed in Table 2. The reaction was performed in a 20 μL volume that contained 10 μL of 2 × Taq Master Mix (Vazyme, P112-01), 1 μL each of the different templates described above, 2 μL primer sets (final concentration of each primer is 100μM), and 7 μL of ddH2O. The procedure was as follows: 95 °C for 3 min, followed by 34 cycles of 95 °C for 15 s, 47 °C for 15 s, and 72 °C for 15 s, with a final extension at 72 °C for 5 min. The PCR fragments obtained were cloned in pMD19-T Vector (TaKaRa), and different clones were analyzed by conventional bidirectional sanger sequencing. The sequencing results were then assembled by the DNASTAR Lasergene software 15.1 package and processed by the online tool Clustal Omega (https://www.ebi.ac.uk/Tools/msa/clustalo/, accessed on 15 January 2023). Multiple-alignment and phylogenetic analyses of the full-length CDV-H, GPRV-VP3, and CPV-2-VP2 genes were then conducted with representative viral sequences from Genbank using the neighbor-joining method in MEGA X, respectively.

## 3. Results

### 3.1. Establishment of a Multiplex PCR (mPCR) Method That Allows Detection of CDV, CPV-2 and GPRV

**Figure 2:**
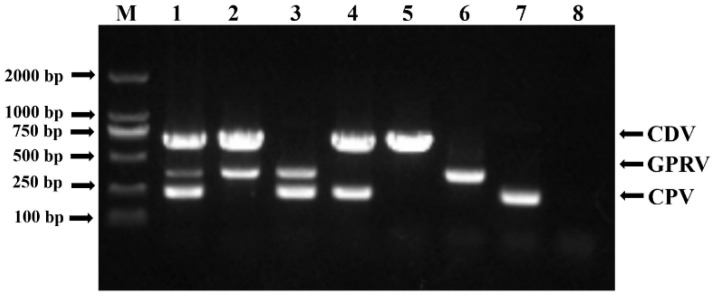
Specificity detection using the mPCR method. M: DL 2000 DNA Marker; 1: CDV + GPRV +CPV-2; 2: CDV + GPRV; 3: GPRV + CPV-2; 4: CDV +CPV-2; 5: CDV; 6: GPRV; 7: CPV-2; 8: negative control.

**Figure 3:**
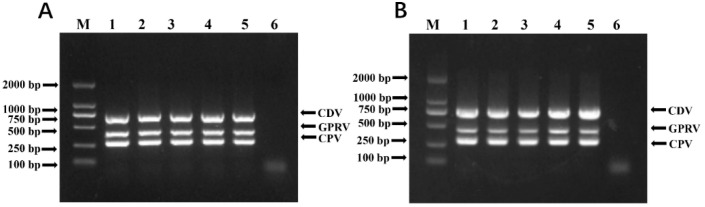
Optimal annealing temperatures for mPCR method. (**A**) M: DL 2000 bp DNA Marker; 1–5: gradient annealing temperatures were 43 °C, 44 °C, 45 °C, 46 °C, and 47 °C, respectively; 6: negative control. (**B**) M: DL 2000 bp DNA Marker; 1–5: gradient annealing temperatures were 48 °C, 49 °C, 50 °C, 51 °C, and 52 °C, respectively; 6: negative control.

Through the cautious design of primers and the adjustment of PCR reaction conditions, the specific detection of the three viruses listed above could be achieved. Plasmid-based viral templates, alone or in combination, were first used to verify the mPCR setting, while the products were separated on a 1.0% agarose gel (Figure 2). The results indicated that specific PCR bands (757 bp for CDV, 432 bp for GPRV, and 293 bp for CPV-2) were clearly visible in each group without unspecific amplification. The authenticity of each PCR product was further confirmed by T-A cloning and sequencing analysis. Meanwhile, to test mPCR’s compatibility with different DNA polymerases, we tested the mPCR system at different annealing temperatures. As shown in Figure 3, clear PCR bands were equally visible with annealing temperatures ranging from 43 °C to 52 °C, and a temperature of 48 °C was used for further analysis.

### 3.2. Sensitivity of the Established mPCR Method

**Figure 4:**
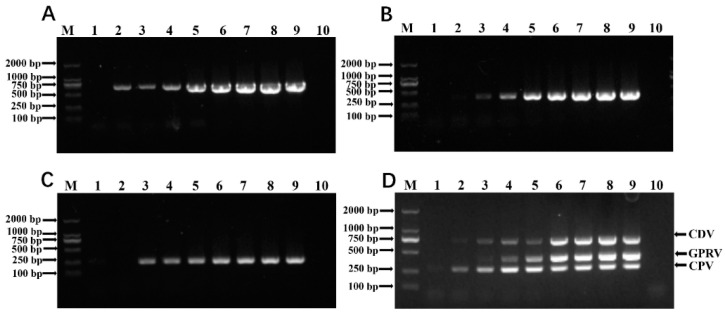
The sensitivity of the established mPCR method. The three single plasmids (pMD-CDV-N, pMD-GPRV-NSP1 and pMD-CPV-2-NS), diluted from 1 × 10^8^ to 1 × 10^1^ copies/μL, and the mixed plasmids (pMD-CDV-N/pMD-GPRV-NSP1/pMD-CPV-2-NS), diluted from 1 × 10^8^ to 1 × 10^1^ copies/μL, were used to determine the minimum detection limit of the mPCR method. (**A**) The sensitivity of pMD-CDV-N; (**B**) the sensitivity of pMD-GPRV-NSP1; (**C**) the sensitivity of pMD-CPV-2-NS; (**D**) the sensitivity of pMD-CDV-N/pMD-GPRV-NSP1/pMD-CPV-2-NS. M: DL 2000 DNA Marker; 1–9: 1 × 10^1^–1 × 10^8^ copies/μL; 10: negative control.

To explore the detection limit of the established mPCR method, different concentrations of plasmids, alone or in combination, were employed as templates for the mPCR reaction. The results showed that the minimum detection limit for the mPCR was 1 × 10^3^ viral copies of each virus when the mixed plasmids were used as the template (Figure 4); however, when only one plasmid was used as template, the sensitivity of the method was higher with minimum detection limits for CDV of 1 × 10^1^ viral copies, and for GPRV and CPV-2 of 1 × 10^2^ viral copies, respectively (Figure 4).

### 3.3. Detection of Viral Co-Infections in Giant Panda from Fecal Samples

**Figure 5:**
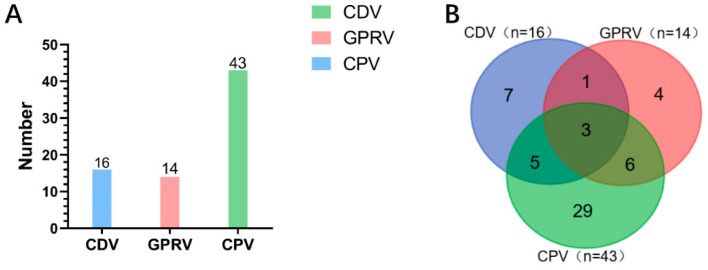
The evaluation of (sub)clinical samples. The detection of viral co-infection in giant panda fecal sample by mPCR (n = 218). (**A**) The number of positive samples is graphed for the mPCR method performed. (**B**) The overlap of viral infections is shown in a Venn diagram.

Using the mPCR method established, a total of 218 hiant panda fecal samples were analyzed to detect viral infection. As indicated in Figure S1, the PCR electrophoresis pattern of the clinical samples was similar to that obtained with plasmid templates, where clear-cut bands were visible for each virus and each possible combination, and occurrences of bands with other sizes were unseen throughout the detection of all samples. The analysis of (sub)clinical samples indicated that 7.34% (16 out of 218), 6.42% (14 out of 218), and 19.72% (43 out of 218) of the samples were positive for CDV, GPRV, and CPV-2, respectively (Figure 5A). Venn analysis revealed the true complex nature of viral co-infections. As shown in Figure 5B, over half of CDV (n = 8)- or GPRV (n = 9)-positive samples were also positive for CPV-2, while one CP-2V-negative sample was positive for both CDV and GPRV. Noticeably, 1.38% of the total samples (3 out of 218) were positive for all three viruses, indicating that superinfections might have occurred in those pandas at the time of sampling.

### 3.4. Consolidation and Validation of mPCR Results Through Regular PCR and Phylogenetic Analysis

**Figure 6:**
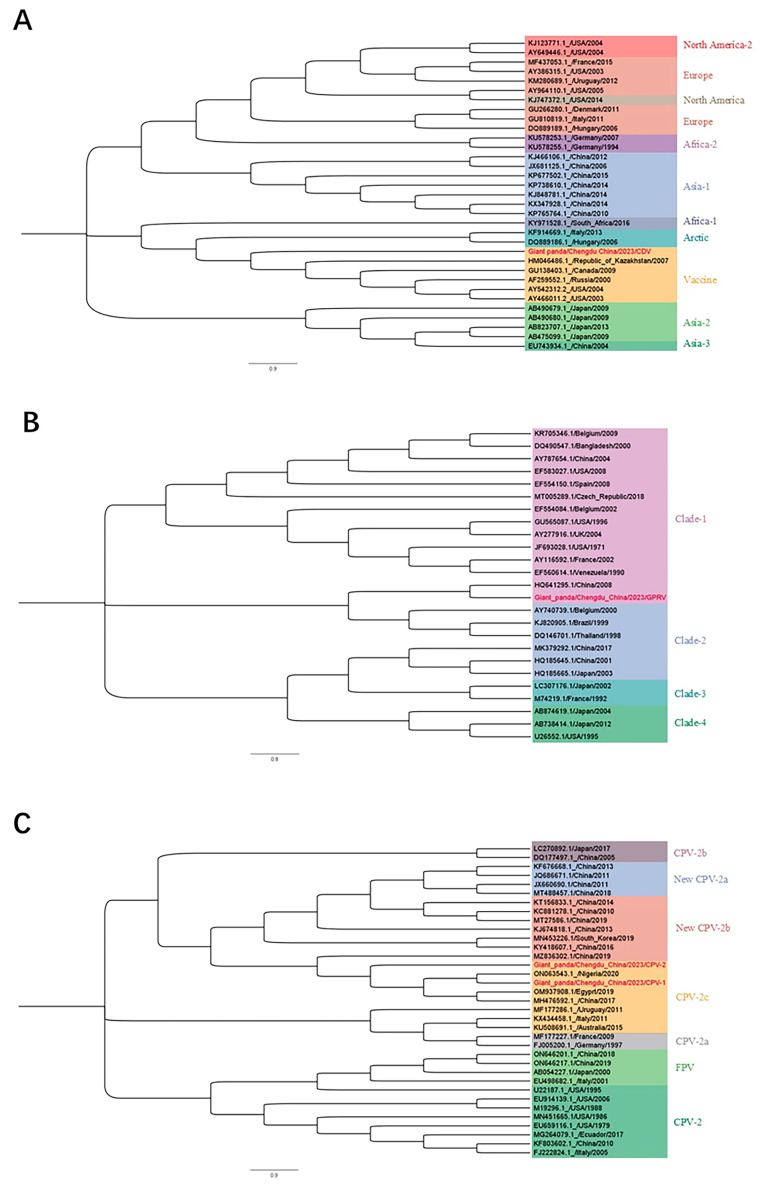
(**A**) Phylogenetic analysis of canine distemper virus (CDV) strains based on H gene sequences; (**B**) phylogenetic analysis of rotavirus (RV) strains based on VP3 gene sequences; (**C**) phylogenetic analysis of canine parvovirus (CPV-2) strains based on VP2 gene sequences. The sequences obtained in the present study are marked in red.

To further verify the specificity of the mPCR results, we designed primers to amplify the full-length sequence of the CDV H gene, the GPRV VP3 gene, and the CPV-2 VP2 gene, while full sequence information is detailed in Table 2. Regular PCR analyses were performed on randomly selected positive samples (n = 10), with the exception of the samples (n = 3) that were positive for all three viruses in the mPCR analysis. The results of the mPCR analysis were well correlated with routine PCR regarding sample positivity, while the sequencing analysis of routine PCR products converged to one or two represented sequences per virus, namely, n = 1 for CDV, n = 1 for GPRV, and n = 2 for CPV-2. Next, phylogenetic analysis was performed with the complete gene sequences obtained. Figure 6 shows the viral sequence clades with major field isolates in dogs (CDV and CPV-2) or pigs (GPRV) that are prevalent in Chinese companion animals or the breeding industry.

## 4. Discussion

Ever since it was first developed and optimized in the early 1980s [19,20], the PCR test has been a powerful tool in molecular biology [21,22]. The utilization of PCR in different ways permits the rapid and efficient recognition of DNA or RNA sequences of various origins, and benefits relevant fields of both fundamental research and practical application. Here, in the current study, we established a multiplex PCR (mPCR) method that allows the detection of three viruses, either DNA- or RNA-based, simultaneously in giant pandas. The results indicate that the mPCR method is steadfast, with matter-of-fact outcomes from clinical fecal samples that can easily be interpreted.

Conventionally, the mPCR method is considered to have relatively reduced sensitivity and specificity, as multiple PCR reactions occur at the same time, with an inherent risk of competing for polymerases within reactions, and because of the cross-reactivity of the primer pairs in use. Nevertheless, through the development of bioinformatic databases and reagents for molecular biology research, the reliability of mPCR can be largely improved in both areas [23]. The careful design of primers, including the use of homology analysis against pathogens and host sequences, could efficiently avoid the occurrence of cross-reactions or nonspecific reactions with other pathogens or host genetic materials [24]. In the meantime, the fast development of commercial polymerases also enables the robust competence of the PCR reaction, even if multiple primers are involved. In the present study, the established mPCR method ran smoothly across all clinical samples, with clear indications of positive or negative outcomes without the ambiguous amplification that results in unexplainable PCR bands. Meanwhile, the sensitivity of the results was 1 × 10^1^ (CDV) or 1 × 10^2^ (CPV-2 and GPRV) viral copies, which was already comparable to that of the traditional PCR. As such, our observations indicated that the established mPCR technique is reliable and can be reinforced in routine molecular diagnostics of viral infections in giant pandas.

The fast development of viromics research and bioinformatics techniques benefited the field of epidemiology greatly, where viral host spectrums were identified via advanced sequencing [25,26]. Newly identified pathogens and their associated diseases demand effective molecular diagnostic methods, especially ones accessible to laboratories with fewer facilitaties. As a conventional technique, the mPCR method has the inherent advantage of being cost-effective, easily operated, and less time-consuming [27,28,29]. Therefore, mPCR at present could serve as a part of a fast-reacting system that allows for rapid detection under circumstances such as an acute outbreak and provides first-hand etiological information that could assist clinical treatment or epidemiological policy making.

The cross-species transmission of various pathogens poses a risk to endangered species such as giant pandas. Contact with humans or other animals, albeit often well protected, also leads to an inevitable consequence of reverse zoonosis or horizonal transmission, via which bacterial or viral infectious agents are introduced to the giant panda population [30,31,32]. Utilizing the established mPCR method, we showed that CDV, GPRV, and CPV-2 were detected in giant pandas, and that co-infection with two or three viruses was commonly observed. The observation was further consolidated with phylogenetic analysis of the matching full-length sequences of mPCR targets. No significant correlation was spotted between symptoms of sampled animals and virus detection in the present study, indicating that infection with these viruses was mostly subclinical, and that acute phases of infection might be neglected or wrongly interpreted. Among the three viruses evaluated, CPV-2 was the most frequently (19.72%) detected in Giant pandas during this investigation. CPV-2 and related viruses of species *Protoparvovirus Carnivoran 1* can infect a wide range of domestic and wild carnivores, which leads to their worldwide distribution with a broad host spectrum [33,34]. Noticeably, due to the close genetic relationship between CPV and feline panleukopenia virus (FPV) in their VP2 sequences and the entire genome [35,36], our method (or any approach without DNA sequencing) cannot differentiate between infection with CPV-2 and FPV, the latter of which was recently reported as being able to infect giant pandas [37]. Further analysis of the full-length VP2 sequences indicated that all amplified parvoviral sequences belong to CPV-2, showing that CPV-2 is the main component of *Protoparvovirus Carnivoran 1* in giant pandas, but the potential threats of FPV transmission in giant pandas should not be neglected. Meanwhile, a smaller but unneglectable number of samples are positive for CDV or GPRV, or both. To this point, recurrent epidemiological surveillance is essential to monitor possible increased transmission of the three virus’s infection in giant pandas and possibly other wild animal species.

In conclusion, we established and validated an mPCR method that allows the rapid and accurate detection of CPV-2, CDV, and GPRV infection. The complex nature of spatial viral infection in giant pandas was revealed via this method, and the key observations justify further investigation on the infection mechanisms of those viruses from both epidemiological and etiological perspectives.

## Figures and Tables

**Table 1:** Primers used for the mPCR method.

**Virus**	**Primer Name**	**Target**	**Sequence (5′-3′)**	**PCR Products (bp)**
CDV GPRV CPV-2	CDV-N-F CDV-N-R GPRV-NSP1-F GPRV-NSP1-R CPV-NS-F CPV-NS-R	N NSP1 NS1	GCATGTCATTATAGTCCTAATCC AAGAGCCGGATACATAGTT AATGATACATGGAGACCATCA AGTCTGATTAACATTGTTCATGT ACAACGTCCAACTAAATGGA ACAATGCCAGCCTTGAT	757 432 293

**Table 2:** Primers used for the full-length gene sequences.

Virus	Primer Name	Target	Sequence (5′-3′)	PCR Products (bp)
CDV GPRV CPV-2	CDV-H-F1 CDV-H-R1 CDV-H-F2 CDV-H-R2 GPRV-VP3-F1 GPRV-VP3-R1 GPRV-VP3-F2 GPRV-VP3-R2 CPV-VP2-F CPV-VP2-R	H VP3 VP2	TTAGGGCTCAGGTAGTCCA TCTACACACAAGGAAGCCA ACTCCAGACAACCAACTAT CTAAGTCCAATTGAAATGTGT GGCTATTAAAGCAGTACTAG AAGAATGTCCAGTCATAGA CCAATGATTACATAGTAGC CATCATGACTAGTGTGTTAAG CCACCACCTCATATTTTCAT CCTTCTAAATCCTATATCAAGTACAA	915 1063 1581 1177 1936

## Data Availability

Data are contained within the article and Supplementary Material.

## Supplementary Materials

The following supporting information can be downloaded at: https://www.mdpi.com/article/10.3390/vetsci12020081/s1, Figure S1: Detection of PCR electrophoretograms of giant panda samples by mPCR.; Table S1: Viruses able to infect giant panda [38,39,40,41,42,43,44,45,46,47,48,49,50,51].
