# Supplementary material for: The Detection of Mixed Infection with Canine Parvovirus, Canine Distemper Virus, and Rotavirus in Giant Pandas by Multiplex PCR
Source: Vet Sci. 2025 Jan 23;12(2):81. doi: 10.3390/vetsci12020081 (PMC11860260; doi:10.3390/vetsci12020081)
Supplement: Supplementary file 1 [file vetsci-12-00081-s001.zip › vetsci-3389132-supplementary.pdf]

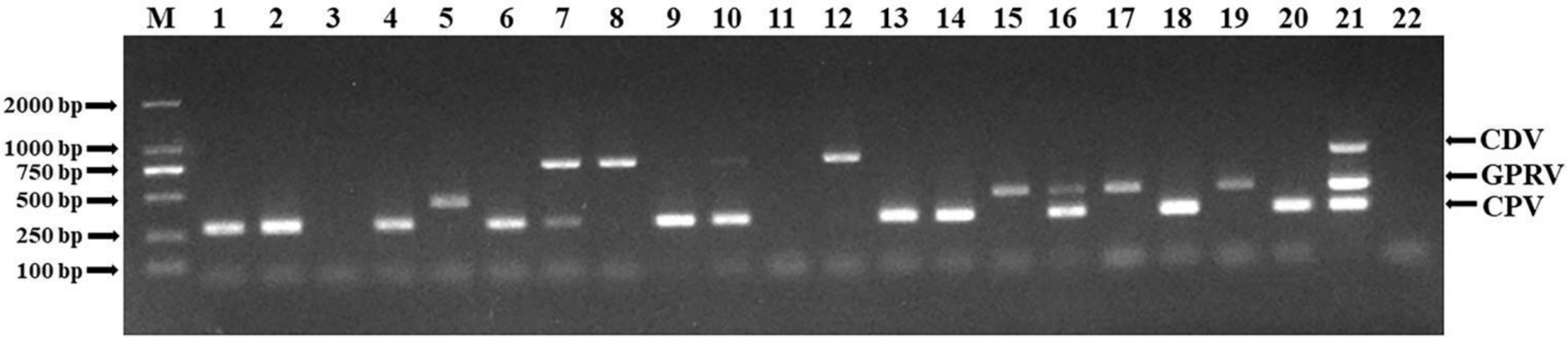

Figure S1. Detection of PCR electrophoretograms of giant panda samples by mPCR. M: DL 2000 DNA Maker; 1-20: Clinical samples; 21: Positive control; 22: Negative control.

Table S1. Viruses able to infect giant panda.

**dsDNA**		**ssDNA**		**dsRNA**		**ss (+) RNA**		**ss (-) RNA**		**ssRNA-RT**
**Ackermannviridae**[1]		**Anelloviridae**[2]		**Totiviridae**[1]		**Leviviridae**[1]		**Arenaviridae**[2]		**Retroviridae**[2]
**Drexlerviridae**[1]		**Microvirida**[1]		**Partitiviridae**[1]		**Iflaviridae**[1]		**Rhabdoviridae**[2]	Rabies virus[3]	
**Myoviridae**[1]		**Genomoviridae**[1, 2]		**Hypoviridae**[1]		**Virgaviridae**[1]		**Paramyxoviridae**[2]	Canine Distemper virus[16-19]	
**Siphoviridae**[1]		**Geminiviridae**[1]		**Endornaviridae**[1]		**Tombusviridae**[1]			Canine Parainfluenza virus[20]	
**Phycodnaviridae**[1]		**Nanoviridae**[1]		**Caulimovirida**[1]		**Picornaviridae**[1, 2]		**Orthomyxoviridae**[2]	Influenza virus[6,18,21]	
**Lridoviridae**[1]		**Circoviridae**[1, 1]	Circovirust[5]	**Reoviridae**[2]	Giant Panda Rotavirus[5,10-12]	**Nodaviridae**[1]				
**Adenoviridae**[2]	Canineadenovirus[3, 4]	**Parvoviridae**[1]	Feline Parvovirus[6, 7]			**Marnaviridae**[1]				
			Canine Parvovirus[3,4,6,8]			**Luteoviridae**[1]				
		**Papillomaviridae**[2]	Polyomavirus[9]			**Coronaviridae**[1]	Canine Coronavirus[3,8,13,14]			
						**Hepatitis E Viridae**[2]	Hepatitis E virus[15]			
						**Hepe-like virus**[1]				
						**Gammaflexiviridae**[1]				
						**Dicistroviridae**[1]				
						**Carmotetraviridae**[1]				
						**Betaflexiviridae**[1]				

Supplementary references

[1] NING S, LU X, ZHAO M, et al. Virome in Fecal Samples From Wild Giant Pandas (Ailuropoda Melanoleuca) [J]. Frontiers in Veterinary Science, 2021, 8.

[2] ZHANG W, YANG S, SHAN T, et al. Virome comparisons in wild-diseased and healthy captive giant pandas [J]. Microbiome, 2017, 5(1): 90.

[3] QIN Q, LI D, ZHANG H, et al. Serosurvey of selected viruses in captive giant pandas (Ailuropoda melanoleuca) in China [J]. Vet Microbiol, 2010, 142(3-4): 199-204.

[4] GUO L, YANG S L, CHEN S J, et al. Identification of canine parvovirus with the Q370R point mutation in the VP2 gene from a giant panda (Ailuropoda melanoleuca) [J]. Virol J, 2013, 10: 163.

[5] DAI Z, WANG H, FENG Z, et al. Identification of a novel circovirus in blood sample of giant pandas (Ailuropoda melanoleuca) [J]. Infect Genet Evol, 2021, 95: 105077.

[6] ZHAO M, YUE C, YANG Z, et al. Viral metagenomics unveiled extensive communications of viruses within giant pandas and their associated organisms in the same ecosystem [J]. The Science of the total environment, 2022, 820: 153317.

[7] YI S, LIU S, MENG X, et al. Feline Panleukopenia Virus With G299E Substitution in the VP2 Protein First Identified From a Captive Giant Panda in China [J]. Front Cell Infect Microbiol, 2021, 11: 820144.

[8] MAINKA S A, QIU X, HE T, et al. Serologic survey of giant pandas (Ailuropoda melanoleuca), and domestic dogs and cats in the Wolong Reserve, China [J]. Journal of wildlife diseases, 1994, 30(1): 86-9.

[9] QI D, SHAN T, LIU Z, et al. A novel polyomavirus from the nasal cavity of a giant panda (Ailuropoda melanoleuca) [J]. Virol J, 2017, 14(1): 207.

[10] YANG R, WANG C, YAN Q. Research progress of giant panda rotavirus strain CH-1 [J]. Chinese Journal of Zoonoses, 2018, 34(11): 1040-3. (in Chinese)

[11] SU X, LI L, YAN X, et al. Establishment and application of giant panda rotavirus PCR detection method [J]. Acta Theriologica Sinica, 2021, 41(03): 254-60. (in Chinese)

[12] WANG C, YAN Q, ZHANG Z, et al. Isolation and identification of rotavirus from giant panda cubs[J]. Acta Theriologica Sinica, 2008, (01): 87-91. (in Chinese)

[13] GAO F S, HU G X, XIA X Z, et al. Isolation and identification of a canine coronavirus strain from giant pandas (Ailuropoda melanoleuca) [J]. J Vet Sci, 2009, 10(3): 261-3.

[14] JUN Q, XIAN-ZHU X, SONG-TAO Y, et al. Serological survey on canine coronavirus antibodies in giant pandas by virus neutralization test [J]. Journal of forestry research, 2004, 15(4): 295-7.

[15] ZHANG C, DING Y, YAN H, et al. Survey of Hepatitis E Virus Infection in Giant Panda [J]. Chinese Journal of Wildlife. 2013: 323-6.10.19711/j.cnki.issn2310-1490.2013.06.003 (in Chinese)

[16] FENG N, YU Y, WANG T, et al. Fatal canine distemper virus infection of giant pandas in China [J]. Sci Rep, 2016, 6: 27518.

[17] ZHAO N, LI M, LUO J, et al. Impacts of canine distemper virus infection on the giant panda population from the perspective of gut microbiota [J]. Sci Rep, 2017, 7: 39954.

[18] GENG Y, SHEN F, WU W, et al. First demonstration of giant panda's immune response to canine distemper vaccine [J]. Dev Comp Immunol, 2020, 102: 103489.

[19] HVISTENDAHL M. Endangered species. Captive pandas succumb to killer virus [J]. Science (New York, NY), 2015, 347(6223): 700-1.

[20] GUO L, LI Z, YANG S, et al. Priamry research and establishment of Rabies virus RT-PCR detection method [J]. Proceedings of 2012 Annual Conference of Sichuan Animal Husbandry and Veterinary Society, 2012: 387-91 (in Chinese)

[21] LI D, ZHU L, CUI H, et al. Influenza A(H1N1)pdm09 virus infection in giant pandas, China [J]. Emerg Infect Dis, 2014, 20(3): 480-3.
